# Interleukin-22 level is negatively correlated with neutrophil recruitment in the lungs in a *Pseudomonas aeruginosa* pneumonia model

**DOI:** 10.1038/s41598-017-11518-0

**Published:** 2017-09-08

**Authors:** Alexis Broquet, Cédric Jacqueline, Marion Davieau, Anissa Besbes, Antoine Roquilly, Jérôme Martin, Jocelyne Caillon, Laure Dumoutier, Jean-Christophe Renauld, Michèle Heslan, Régis Josien, Karim Asehnoune

**Affiliations:** 1 Laboratoire UPRES EA3826 « Thérapeutiques cliniques et expérimentales des infections », IRS2 – Nantes Biotech, Université de Nantes, Nantes, France; 2 CHU Nantes, Pôle anesthésie réanimations, Service d’anesthésie réanimation chirurgicale, Hôtel Dieu, Nantes, F-44093 France; 3 Centre de Recherche en Transplantation et Immunologie UMR1064, INSERM, Université de Nantes, Nantes, France; 4 Institut de Transplantation Urologie Néphrologie (ITUN), CHU Nantes, Nantes, France; 5 Laboratoire d’Immunologie, CHU Nantes, Nantes, France; 6 Ludwig Institute for cancer Research and Institut de Duve, Université Catholique de Louvain, B-1200 Brussels, Belgium

## Abstract

*Pseudomonas aeruginosa* is a major threat for immune-compromised patients. Bacterial pneumonia can induce uncontrolled and massive neutrophil recruitment ultimately leading to acute respiratory distress syndrome and epithelium damage. Interleukin-22 plays a central role in the protection of the epithelium. In this study, we aimed to evaluate the role of interleukin-22 and its soluble receptor IL-22BP in an acute *Pseudomonas aeruginosa* pneumonia model in mice. In this model, we noted a transient increase of IL-22 during *Pseudomonas aeruginosa* challenge. Using an antibody-based approach, we demonstrated that IL-22 neutralisation led to increased susceptibility to infection and to lung damage correlated with an increase in neutrophil accumulation in the lungs. On the contrary, rIL-22 administration or IL-22BP neutralisation led to a decrease in mouse susceptibility and lung damage associated with a decrease in neutrophil accumulation. This study demonstrated that the IL-22/IL-22BP system plays a major role during *Pseudomonas aeruginosa* pneumonia by moderating neutrophil accumulation in the lungs that ultimately leads to epithelium protection.

## Introduction

Pneumonia induced by *Pseudomonas aeruginosa* (PA), a Gram-negative opportunistic bacteria, is a major threat for immune-compromised patients^1^. During infection, the host must activate a robust but adapted immune response against the pathogen while protecting the integrity and the functionality of the lungs. In the early period of pulmonary infection, there is massive polymorphonuclear neutrophil (PMN) recruitment generating oedema and tissue damage through the generation of an oxidative burst and pro-inflammatory microenvironment. Deregulated and overwhelming activation of PMN can lead to destruction of the alveolar-capillary barrier and to acute respiratory distress syndrome (ARDS)^2^.

Interleukin (IL)-22 is a member of the IL-10 superfamily and is currently described as the cytokine of epithelium protection. Although RORγT_pos_ type-3 Innate Lymphoid Cells (ILC3) are characterized by their ability to produce IL-22^3^, other cells such as NK cells^4^, alveolar macrophages^5^ and neutrophils^6^ have been suspected of producing IL-22^7^. Owing to an almost restricted expression of the membrane IL-22 receptor (IL-22R) to epithelial cells^8^, IL-22 exerts crucial functions in regulating epithelial biology^9^. Based on antimicrobial peptides (AMP) and mucus production induction, the actions of IL-22 have been shown to be significant in fighting a number of extracellular bacteria and fungi at barrier surfaces of the gut and the lungs^10–12^. For example, IL-22 expression induced by C. *albicans* exposure in the lungs is protective against secondary PA infection^13^. In addition, IL-22 displays significant tissue-protective properties and supports epithelium wound healing and regeneration after injury by controlling epithelial cell proliferation, survival and differentiation^14–16^. Overall, these data suggest that IL-22 could limit epithelial lung injury during ARDS, especially when secondary to acute bacterial infection.

In contrast, there are indications that IL-22 could also contribute to pathogenic epithelial-destructive inflammation by stimulating the release of matrix metalloproteases and PMN-recruiting chemokines and by promoting aberrant epithelial cell proliferation and differentiation^17–19^. This duality of IL-22 functions during inflammation probably reflects the significance of tissue context in determining the balance of IL-22 protective vs. deleterious actions on epithelial cells. In support of this idea, Sonnenberg *et al*. previously showed that during bleomycin-induced acute lung injury, the tissue-protective effects of IL-22 are overwhelmed by pro-inflammatory properties owing to the synergistic actions with IL-17 to recruit PMNs^20^. Therefore, it is not surprising that IL-22 possesses a specific system of regulation that is an IL-22 Binding Protein (IL-22BP), a secreted, soluble and specific inhibitor^21^ which we previously showed to be produced by a specific subset of immature dendritic cells in rodent gut that negatively regulates the protective actions of IL-22 during DSS-induced acute colitis^22, 23^.

There are indications that both IL-22 and IL-22BP are produced in the bronchoalveolar fluid of ARDS patients^24^. However, it has remained unclear if, in this condition, IL-22 exerts protective actions on the epithelial cells that are blocked by IL-22BP and vice versa. Given the critical importance of epithelial injury in determining the outcome of ARDS patients^25^, deciphering the role of the IL-22/IL-22R/IL-22BP axis could provide a major new therapeutic perspective.

In this study, we showed that IL-22 neutralisation led to an increase in PMN recruitment and lung lesions. Increased IL-22 levels (administration of recombinant IL-22 (rIL-22) or neutralisation of IL-22BP) induced a decrease in PMN recruitment and lung lesions. Taken together, these data demonstrated a protective role of IL-22 through its ability to modulate PMN recruitment.

## Materials and Methods

### Mice, bacteria strain and cell line

Eight-to-ten-week-old pathogen-free RjOrl:SWISS mice (weight, 29–32 g) were purchased from Janvier Laboratories (Le Genest Saint Isle, France). The mice were maintained on a 12-hour light/dark cycle with access to food and water ad libitum. The animals were treated in accordance with institutional policies and the guidelines stipulated by the animal welfare committee. The Ethics Committee for Animal Experiments of the Loire Department (University of Angers, C2EA-06) approved all of the animal experiments in this study. PA strain PAO1 was grown as previously described^26^ and the inoculum was calibrated by nephelometry (2 × 10^8^ CFU/mL). A549 cell line was obtained from Dr Vié (Nantes, France) and was cultured in RPMI medium complemented with 10% foetal bovine serum and 5mM L-glutamin. Cells were seeded at a density of 500,000 cells/mL in 24-well plates and cultivated at 37 °C with 5% CO_2_ for 3 days until the time of the experiment.

### Pneumonia model and neutralising antibody administration

Pneumonia was induced as previously described^26^. For the IL-22 and IL-22BP neutralisation experiments, anaesthetised mice were subjected to a single anti-IL22, anti-IL-22BP or isotype control (mouse IgG2a. BioLegend) antibody administration i.v. the day before the induction of pneumonia (50 μg/mouse). Neutralising anti-IL22 and IL-22BP (clone AM22BP.4) antibodies were provided by JC Renauld^27^.

### Bacteriological assessment of lung and evaluation of systemic dissemination

Lungs were removed and homogenised in 1 mL of saline buffer (Mixer Mill MM 400, Retsch Inc., Newtown, PA, USA) and used for quantitative cultures on Mueller-Hilton agar for 24 hours at 37 °C. Serial dilutions were performed and viable counts after 24 hours of incubation were expressed as the mean ± SD log_10_ Colony Forming Unit (CFU) per gram of organ.

### Determination of cytokine levels in the lungs by ELISA

Immediately after removal, weighed lung samples were mechanically homogenised in cold lysis buffer (1X phosphate buffered saline [PBS, pH 7.4], 0.1% Triton X-100) containing 1 mM protease inhibitor cocktail (Sigma, St Quentin Fallavier, France). CXCL-2 (MIP-2a), interleukin (IL)-1β, interleukin (IL)-6, IL22 and Tumour Necrosis Factor (TNF)-α concentrations in lung homogenates were quantified with ELISA kits according to manufacturer instructions (For CXCL2: R&D Systems, Lille, France; for IL1-β, IL-6, IL22 and TNF-α: eBioscience, France). The protein concentration in each sample was determined using a BCA™ protein assay kit (Pierce, Rockford, IL, USA).

### Bronchoalveolar Fluid (BALF)

Euthanized mice were put in dorsal recumbency and the trachea were exposed. A 22-gauge catheter was inserted in the trachea and the lungs were washed 3 times with 1 mL of cold 0.9% NaCl. Red blood cell counts were determined in BALF through Iris IQ200 select automated system (Beckman Coulter).

### Histology and immunohistochemistry

At 0, 24, 48 or 72 hours of infection, groups of 3 mice were euthanized and both lungs were removed and immediately placed in 4% formalin. Formalin-fixed tissues were processed and stained with haematoxylin and eosin (H&E). For IL-22 and neutrophil staining, anti-IL-22 (polyclonal goat IgG, 2 μg/mL, R&D Systems, Lille, France) and anti-Ly6-G (clone 1A8, 5 μg/mL, Ozyme, Saint Quentin-en-Yvelines, France) antibodies respectively and corresponding isotype control antibodies were used following manufacturer instructions. See suppl. method for detailed information.

### Real-time quantitative RT-PCR

Total RNA was isolated using Trizol reagent (Fisher Scientific) or Qiagen RNeasy Mini Kit according to manufacturer instructions. Reverse transcription was performed using Murine Moloney Leukaemia Virus Reverse Transcriptase (Fisher Scientific) or Superscript First-Strand Synthesis System for RT-PCR (Fisher Scientific), following manufacturer instructions. For gene expression, Power Sybr® Green 2 × reagent was used (Applied Biosystems, Foster City, CA). Real-time PCR was performed using the Viia™ 7 Real Time PCR system (Applied Biosystems). See suppl. method for primer information.

### Statistical analysis

GraphPad prism software (La Jolla, CA. United States) was used for statistical analysis. Continuous non-parametric variables were expressed as median (25^th^–75^th^ percentile). The Kruskal-Wallis test was used for comparisons of multiple groups. Dunn’s multiple comparison test was used as post hoc test for intergroup comparisons. Survival curves were compared with a log-rank test. p < 0.05 was considered to be statistically significant.

## Results

### PA pneumonia induces lung epithelial cell damage

**Figure 1:**
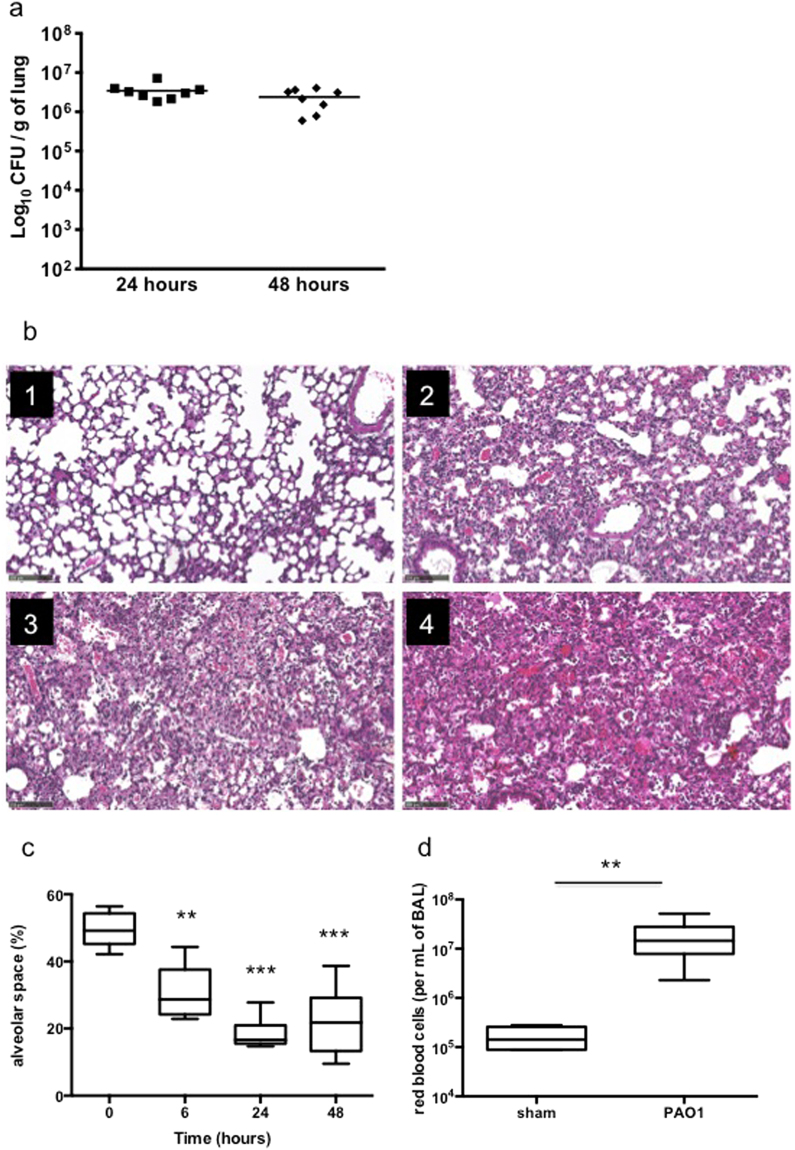
PA induces lung oedema and epithelium damage during pneumonia. (**a**) Pulmonary bacteria loads of mice infected with PA for 24 and 48 hours. Data are representative of two independent experiments (n = 8). (**b**) Lung histological analysis from sham Swiss mice (panel 1) or PA infected mice at 6 (panel 2), 24 (panel 3), and 48hrs (panel 4) of infection. Magnification × 100 (bar = 100 μm). Data are representative of two independent experiments (n = 3). (**c**) Alveolar space quantification by SIOX analysis of histology slides presented in (**b**) (3 mice per slide. 4 fields per slide). (**d**) Red blood cell counts in BALF fluid in sham mice versus 24-hour infected mice. Data are representative of two independent experiments (n = 3). **p < 0.01 and ***p < 0.001.

The PA acute pneumonia model in mice led to the development of severe epithelium damage and lung oedema with stable pulmonary bacteria loads from 24 to 48 hours of infection (Fig. 1a). Compared with non-infected lungs where single-layer cells surround alveoli (Fig. 1b, panel 1), PAO1 infection led to rapid and increasing epithelium cell layer thickening, massive recruitment of immune cells and alveolar septa destruction from 6 hours to 48 hours of infection (Fig. 1b, panels 2–4). Alveolar space evaluation showed a significant decrease in the alveoli compartment consistent with the generation of lung oedema (Fig. 1c). PAO1 pneumonia then induced alveoli haemorrhage in infected-BALF (Fig. 1d).

### Interleukin-22 is transient during PA pneumonia

**Figure 2:**
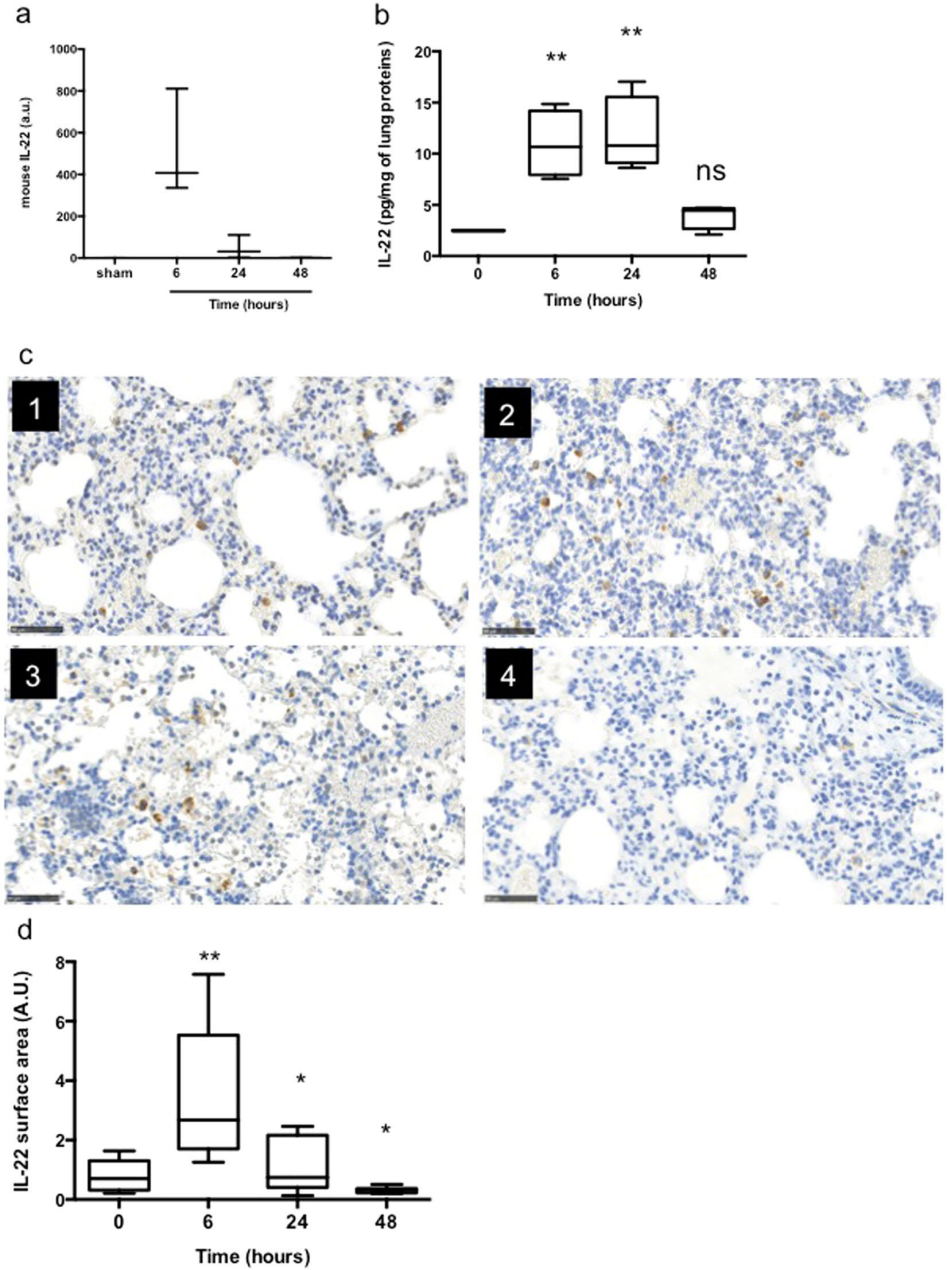
PA induces a transient increase of interleukin-22 in the lungs. (**a**) IL-22 mRNA expression levels in total lungs at different time points of infection. Data are representative of two independent experiments (n = 3 per group). (**b**) IL-22 levels by ELISA in lung homogenates of mice infected at different time points. Boxes represent median (interquartile range). Data are representative of two independent experiments (n = 6 per infected group. n = 3 for the sham group). (**c**) IL-22 IHC of lung section from sham (panel 1) and infected mice at 6 (panel 2), 24 (panel 3) and 48hrs (panel 4). Data are representative of two independent experiments (n = 3 per group). Magnification: × 100. Bar = 100 μm. (**d**) Quantification of IL-22 positive pixels surface area by SIOX analysis of the slides presented in (**c**). Data are representative of two independent experiments (3 mice per group; four fields per slide). *p < 0.05, **p < 0.01, and n.s.: not significant compared with the sham group.

In this context of epithelium damage, we assessed the expression of the interleukin (IL)-22 during PA pneumonia (Fig. 2). PAO1 infection induces a tremendous and transient IL-22 mRNA increase at 6 hours of infection (Fig. 2a). This is correlated with the transient increase of IL-22 protein levels in the lungs of infected mice 6 and 24 hours after the onset of pneumonia (p < 0.01) followed by a drop back to IL-22 basal levels at 48 hours (ns; p = 0.4 compared with the sham group) (Fig. 2b). IL-22 detection on paraffin-embedded lung sections confirmed this transient IL-22 increase (Fig. 2c). IL-22 positive pixel surface quantification revealed that after an initial increase, IL-22 positive staining in the 48-hour infected lungs was significantly decreased compared with the sham group (p < 0.05) (Fig. 2d).

### IL-22 *in vivo﻿* neutralisation increases mice susceptibility to infection

**Figure 3:**
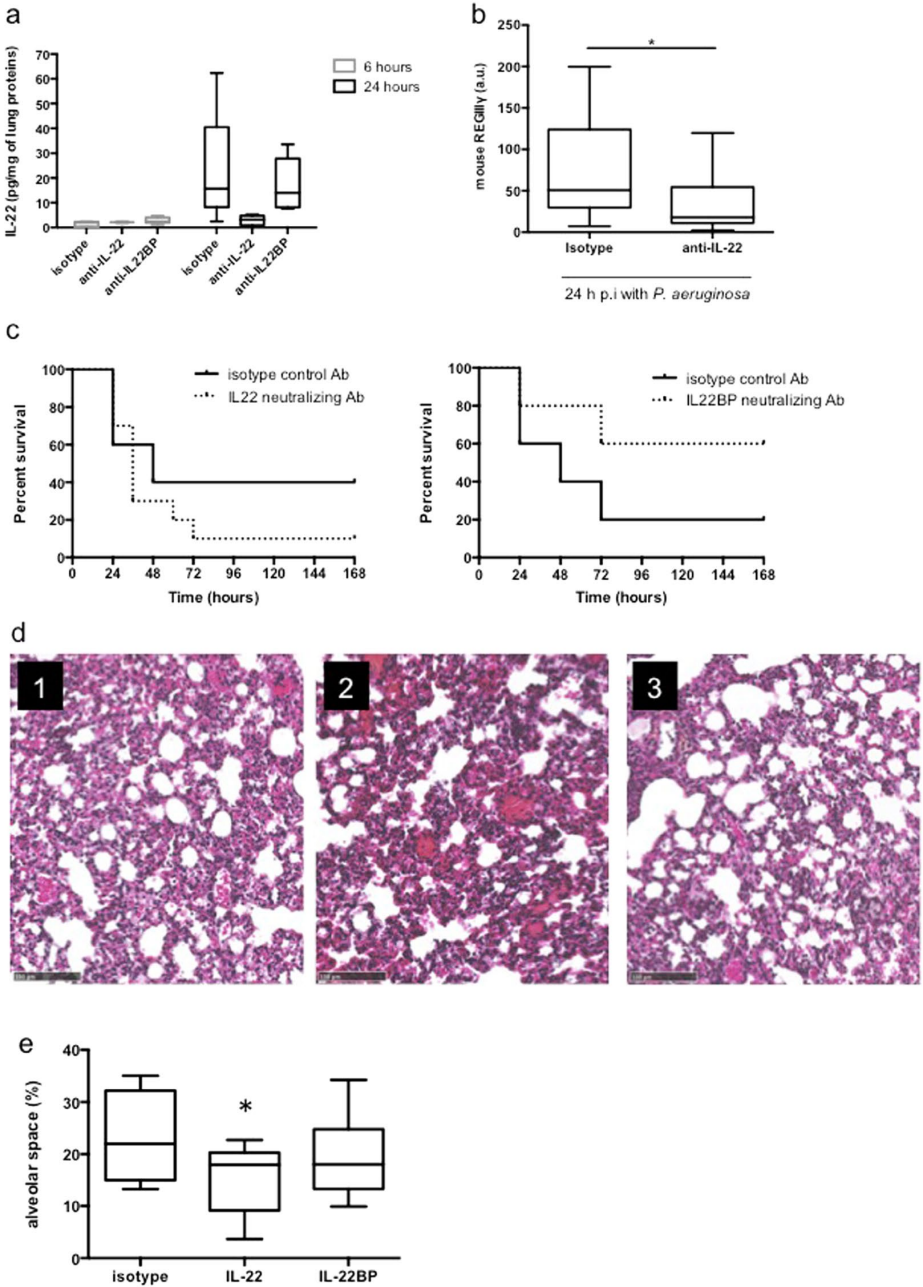
IL-22 neutralisation enhances mice susceptibility and lung damage to PA. (**a**) IL-22 level assessment by ELISA in lung homogenates of IL-22 neutralised mice. Boxes represent median (interquartile range). Data are representative of two independent experiments (n = 6 per infected group). *p < 0.05 and n.s.: not significant compared with the sham group. (**b**) Impact of IL-22 neutralisation on REGIIIγ mRNA expression in total lungs of 6-hour infected mice. Data are representative of 2 independent experiments (n = 5). (**c**) Survival curves of infected mice treated with an isotype control antibody (solid line) or with an IL-22- neutralising antibody (left panel – dashed line) or with and IL22-BP neutralising antibody (right panel – dashed line). Survival rates are expressed as percentage and are representative of 2 independent experiments (anti-IL-22: n = 8 per group; anti-IL-22BP: n = 5 per group). (**d**) Lung histological analysis from infected mice treated with an isotype control antibody (panel 1), an IL-22- neutralising antibody (panel 2) or with an IL-22BP neutralising antibody (panel 3). Magnification × 100. Bar = 100 μm. Data are representative of two independent experiments (n = 3). (**E**) Alveolar space quantification by SIOX analysis of histology slides presented in (**d**) (3 mice per slide. 4 fields per slide). *p < 0.05 compared with the isotype group.

In order to assess the relevance of IL-22 in the context of PA pneumonia, IL-22 and IL-22BP neutralising antibody-based approaches were conducted. Intravenous administration of neutralising IL-22 antibody 18 hours before the induction of pneumonia led to the abolition of the IL-22 protein increase in the lungs of the 24-hour infected mice (Fig. 3a) whereas a slight IL-22 increase, although not significant (p = 0.08), was observed in IL-22BP neutralised mice at 6 hours of infection. To confirm the relevance of IL-22 neutralisation *in vivo*, antimicrobial peptide REGIIIγ mRNA was monitored while IL-22 regulated REGIIIγ expression^11^. IL-22 neutralisation is correlated to a decrease in REGIIIγ mRNA expression in the lungs of infected mice (Fig. 3b). It is of interest to note that IL-22 neutralisation led to enhanced mouse susceptibility to infection (Fig. 3c, left panel. p = 0.04) whereas IL-22BP neutralisation decreased it (Fig. 3c, right panel. p = 0.03). This increased susceptibility in IL-22 neutralised animals was correlated with an increase in lung damage and oedema (Fig. 3d and e. p = 0.03), thereby demonstrating the protective role of IL-22 during PA acute pneumonia.

### IL-22 neutralisation enhances PMN recruitment during infection

**Figure 4:**
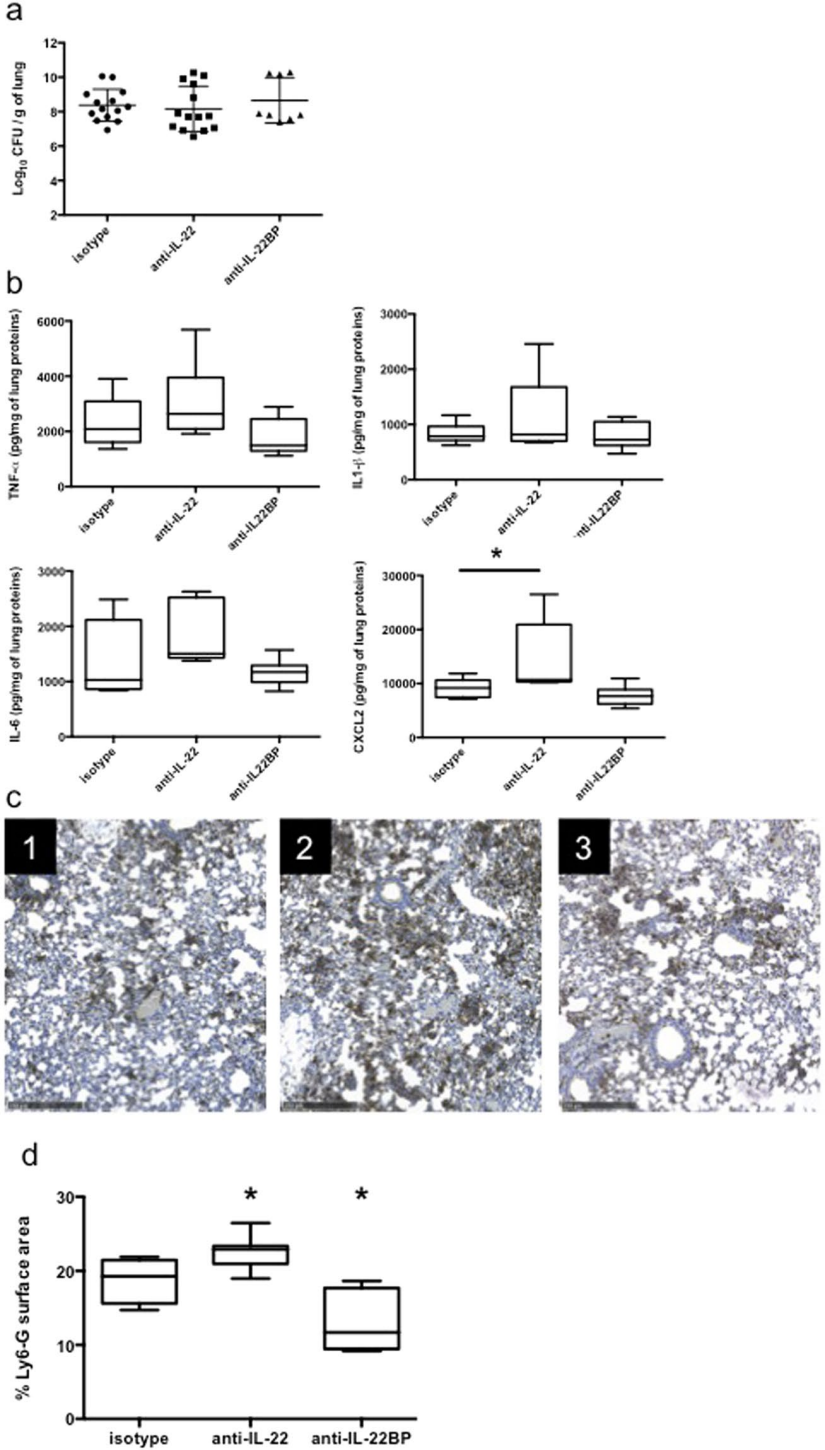
IL-22 neutralisation enhances a PMN-based response during infection. (**a**) Bacterial counts (expressed in log_10_ colony-forming units [CFU]/grams of organ) in the lungs, spleen and kidney of 24-hour infected mice treated with an isotype control antibody or an IL-22 neutralising antibody. Boxes represent median (interquartile range). Data are representative of two independent experiments (n = 6 per group). **p < 0.001. (**b**) TNF-α, IL-1β, IL-6 and CXCL2 concentration assessment by ELISA in lung homogenates of 6-hour infected mice treated with an isotype control antibody or an IL-22 neutralising antibody. Boxes represent median (interquartile range). Data are representative of two independent experiments (n = 6 per group). *p < 0.05. (**c**) Ly6-G IHC of lung section from 6-hour infected mice treated with an isotype control antibody (pan﻿el 1), an IL-22 neutralising antibody (panel 2) or an IL-22BP neutralising antibody (panel﻿ 3). Data are representative of two independent experiments (n = 3 per group). Magnification: × 40. Bar = 250 μm. (**d**) Quantification of Ly6-G positive pixels surface area by SIOX analysis of the slides presented in (**c**). Data are representative of two independent experiments (3 mice per group; four fields per slide). *p < 0.05.

To highlight the impact of IL-22 and IL22-BP neutralisations, bacteria loads and host inflammatory response were assessed. As shown in Fig. 4a, *in vivo* neutralisation of IL-22 prior to infection did not affect pulmonary bacteria loads. Interestingly, IL-22 neutralisation tended to increase the levels of all cytokines tested although only significantly for the chemokine CXCL2 (p < 0.05) (Fig. 4b). CXCL2 (IL-8 human homolog) is known to be central for the recruitment of PMN in the lungs during infection. As shown in Fig. 4c and d, IL-22 neutralisation led to a significant increase in Ly6-G immunostaining (Fig. 4c, panel 2) showing higher PMN recruitment (Fig. 4d, p = 0.03) whereas IL-22BP neutralisation led to a decrease in PMN recruitment (p = 0.05) in the lungs of 6-hour infected mice.

### rIL-22 administration moderates PMN recruitment during infection

**Figure 5:**
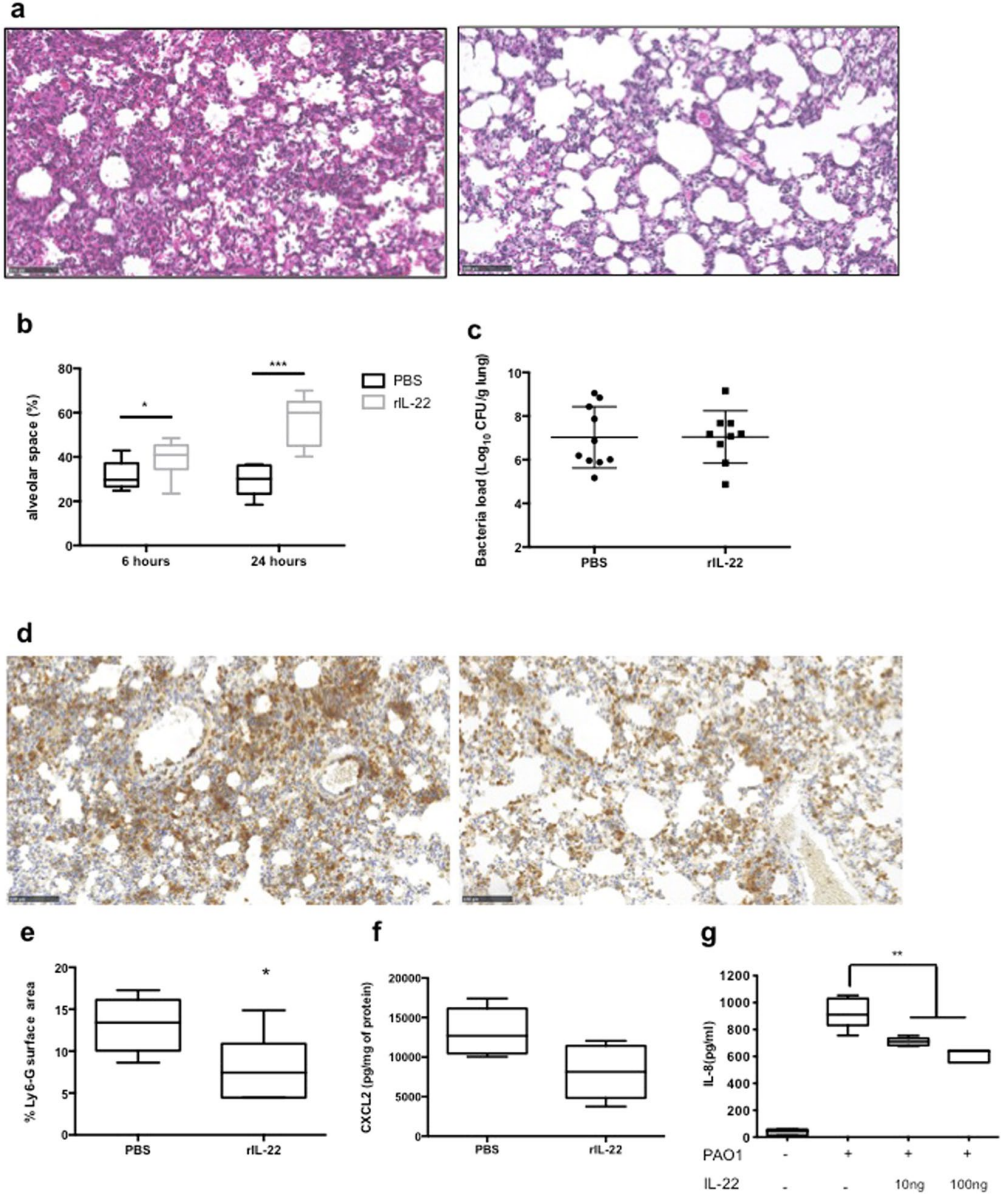
rIL-22 administration during PA infection attenuates lung damage and oedema. (**a**) Histology of 24-hour infected lung PBS-treated (left panel) or rIL-22-treated (right panel) 18hrs before infection. Data are representative of two independent experiments (n = 3 per group). Magnification × 20. Bar = 100 μm. (**b**) Alveolar space quantification by SIOX after rIL-22 administration in 6- and 24-hour infected lungs. Data are representative of two independent experiments (3 mice per group; four fields per slide). *p < 0.05 and ***p < 0.01. (**c**) Pulmonary bacteria loads of 24-hour infected mice treated with rIL-22. Data are representative of two independent experiments (n = 6 per group). (**d**) Ly6-G IHC of lung section from 6-hour infected mice treated with PBS (left panel) or recombinant IL-22 (rIL-22 - right panel) 18 hours before infection. Data are representative of two independent experiments (n = 3 per group). Magnification: × 20. Bar = 100 μm. (**e**) Quantification of Ly6-G positive surface area by SIOX analysis of the slides presented in (**d**). Data are representative of two independent experiments (3 mice per group; four fields per slide). *p < 0.05. (**f**) CXCL2 concentrations assessment by ELISA in lung homogenates of 6hrs infected mice treated with 100ng of rIL-22 or PBS. Boxes represent median (interquartile range). Data are representative of two independent experiments (n = 6 per group). (**g**) IL-8 quantification by ELISA of 6-hour infected A549 cell supernatant treated or not with rIL-22 18hrs before infection. Data are representative of two independent experiments (n = 3 per group). **p < 0.01.

During PA pneumonia, we observed a correlation between the levels of IL-22 in the lungs of infected mice and the histological damage observed. We addressed the impact of the *in vivo* administration of recombinant IL-22 before infection on lung damage after infection. As shown in Fig. 5a, intra-tracheal administration of 100ng of rIL-22 18 hours before the induction of pneumonia greatly decreased epithelial cell damage and lung oedema. In particular, rIL-22 administration significantly attenuated shrinking of the alveolar space during infection at 6 and 24 hours (Fig. 5b, p < 0.05 and p < 0.001 respectively). Since rIL-22 administration did not have an impact on bacteria load during pneumonia (Fig. 5c), we suspected an effect of rIL-22 on the host response. rIL-22 administration led to a moderate but significant decrease in PMN recruitment during infection as shown by Ly6-G IHC (Fig. 5d, right panel) and Ly6G surface staining quantification (Fig. 5e, p = 0.03). Interestingly, rIL-22 administration prior to infection tended to decrease MIP-2 expression in the lungs of infected mice compared with PBS-treated mice (p = 0.17) in contrast with IL-22 neutralisation which led to an increase of CXCL2 expression (Fig. 5f). IL-22 action is restricted to epithelial cells owing to the specific expression of the IL22RA1 receptor chain in these cells. Since IL-22 neutralisation impacted CXCL2 levels in the lungs and PMN recruitment, we explored the ability of rIL-22 to directly modulate IL-8 production on human epithelial A549 cell lines. As displayed in Fig. 5g, rIL-22 incubation on cells 18 hours before infection decreased IL-8 secretion during 6-hour PAO1 infection in a dose-effect manner (p < 0.01) confirming *in vitro* the effect of rIL-22 observed *in vivo*.

## Discussion

In this study, we showed a correlation between the protective role of IL-22 during PA pneumonia and PMN recruitment in the lungs. Mice in which IL-22 had been neutralised displayed aggravated lung damage, increased neutrophilic response and mice susceptibility during infection. On the other hand, mice rescued with rIL-22 administration or in which IL22-BP had been neutralised showed a decrease in pulmonary damage and neutrophilic response.

In agreement with other studies addressing the role of IL-22 in the context of pathogen-induced pulmonary disease^7^, we observed a protective role of IL-22 during acute PA pneumonia in mice. However, in contrast to S.J. Aujla *et al*. in a *K. pneumonia* pneumonia model^10^, pulmonary IL-22 level modulation by exogenous administration or antibody neutralisation did not affect pulmonary bacterial loads compared with the untreated animals. The absence of bacterial burden modification suggests that the protective action IL-22 is not mediated by its direct anti-bacterial properties but rather through the ability of IL-22 to modulate host inflammatory response and susceptibility. To the best of our knowledge, the role of AMP in mucosal immunity in lungs has been poorly studied. It may be hypothesized that like in the gut, RegIII-γ regulates bacterial virulence by maintaining a zone of physical separation between the mucosal surface and bacteria without the need to decrease bacterial burden^28^. RegIII-γ could also interfere with the lung microbiome and decrease the virulence of PA.

IL-22 exhibits pro- or anti-inflammatory properties depending on the environment^29^. In our model, IL-22 acted as an anti-inflammatory molecule since it was correlated with PMN recruitment in the lungs. Tuning appropriate host response, especially PMN recruitment in response to pathogen aggression, is critical for host survival. PMN recruitment and activation during bacterial infection is a double-edged sword^30^. Neutropenic mice display exacerbated susceptibility of PA pneumonia^31^ and neutrophil depletion (by i.v. administration of Ly6-G neutralisation antibody) which led to a fatal lung infection in our model within 12 hours (data not shown). Failure to properly control PMN accumulation following pulmonary infection will contribute to tissue damage and ARDS^26, 30, 32^. In the context of chronic obstructive pulmonary disease (COPD), Guillon *et al*. showed that PMN proteases may alter IL-22 pathways leading to an increase in tissue damage^33^. The current results confirm that PA may alter the outcome of pneumonia through a massive recruitment of PMN in the lungs, thereby enhancing pulmonary lesions. Confirming these data, prophylactic administration of IL-22 correlated with a decrease in CXCL2 levels and PMN accumulation during infection. Although intra-tracheal administration of rIL-22 in the lung suggests a local effect, we cannot exclude systemic spreading of rIL-22 especially to the liver, an organ known to highly express IL22RA1. For example, in a model of pneumococcal pneumonia, G. Trevero-Nunez *et al*. demonstrated that liver-specific IL22RA1 deletion resulted in an increase of bacterial burden in the lungs^34^.

Several studies have pointed out the correlation between CXCL2 expression and disease severity in ARDS^35, 36^. This is consistent with the study by Hoegl *et al*. in which a diminution of CXCL2 levels in the lungs after IL-22 administration in a ventilator-induced lung injury (VILI) model in rat was observed^37^. Moreover, current results show that IL-22-treated A549 human cell line secreted less IL-8 on infection as observed by H.A. Whittington *et al*.^24^. In our model, IL-22 could act as an immune-modulatory cytokine with anti-inflammatory properties. It is consistent with the role of this cytokine in the VILI model of Hoegl *et al*. where the authors found, aside from CXCL2 modulation, an increase of the immune-modulatory protein SOCS3 expression after IL-22 stimulation^37^. Finally, it has been shown that protease IV of PA alters IL-22 dependent lung defence^33^, highlighting the correlation between this pathogen, IL-22 and PMN recruitment.

IL-22 is the only IL10 family member that can interact with a soluble receptor, IL22-BP, a receptor that is highly expressed in the lungs^21, 38, 39^. IL-22 BP is a soluble inhibitor of the IL-22 receptor that could be a major regulator of IL-22 in the context of bacterial pneumonia. Neutralising IL22-BP in our model induced a decreased susceptibility of the mice to the infection, decreased lung damage and PMN recruitment as observed with the administration of recombinant IL-22. These data underlined the significant role of IL-22BP in controlling IL-22 availability during pathological conditions such as acute pneumonia. Our data are in agreement with the G. F. Weber *et al*. study in which administration of recombinant IL-22BP-Fc resulted in an increase in PMN counts in a cecal legation puncture model of microbial sepsis^40^. More recently, we showed in the imiquimod-induced psoriasis model in mice, known to be IL-22 dependent, that *in vivo* neutralisation of IL-22BP with the same neutralising antibody used in the present study led to increased severity of psoriasis-like skin inflammation^41^. Taken together, our data highlighted the role of the IL-22/IL-22BP system during bacterial pneumonia and the need for additional studies to assess its therapeutic interest for patients suffering from bacterial pneumonia or ARDS.

## Electronic supplementary material

Supplementary information
