# Supplementary material for: Interleukin-22 level is negatively correlated with neutrophil recruitment in the lungs in a Pseudomonas aeruginosa pneumonia model
Source: Sci Rep. 2017 Sep 8;7:11010. doi: 10.1038/s41598-017-11518-0 (PMC5591182; doi:10.1038/s41598-017-11518-0)
Supplement: Supplementary file 1 — Supplementary information [file 41598_2017_11518_MOESM1_ESM.doc]

Interleukin-22 level is negatively correlated with neutrophil recruitment in the lungs in a *Pseudomonas aeruginosa* pneumonia model

Alexis Broquet; Cédric Jacqueline; Marion Davieau; Anissa Besbes; Antoine Roquilly, Jérôme Martin; Jocelyne Caillon; Laure Dumoutier; Jean-Christophe Renauld; Michèle Heslan; Régis Josien; Karim Asehnoune.

**Supplemental methods**

**Histological oedema and neutrophil accumulation analysis**

3 slides per condition were scanned with Nanozoomer 2.0–HT C9600 (Hamamatsu, Massy, France), and then captured with the constructer’s NDP viewer software. For lung oedema analysis using the H&E slides, 4 fields per slide at x100 magnification were captured. For neutrophil accumulation in the lungs, 8 fields per slide (Ly6-G IHC) at x200 magnification were captured. Oedema and neutrophil accumulation evaluations were realized on histology slides using the Single Integrative Object eXtraction (SIOX) plugin available in the open-source image analysis software Fiji (www.fiji.sc). For oedema, pixel ratios between alveolar spaces and tissue areas were determined. For IL-22 positive cells and neutrophil accumulation evaluation, ratio between IL-22 and Ly6-G positive pixels, respectively, and picture total pixels were determined. Data were expressed as percent of surface staining (Arbitrary Unit).

**Real-time quantitative RT-PCR**

Primer (Eurofins MWG Operon, Ebersberg, Germany) sequences (5’ to 3’) are as follow. IL-22fwd CCTACATGCAGGAGGTGGTG, IL-22rev AAACAGCAGGTCCAGTTCCC, RegIIIγfwd CAGATATGGCCTGCCAAAAGA, RegIIIγrev TGTTGGGTTCATAGCCCAGTG ; GAPDHfwd  GGTGAAGGTCGGTGTGAACGG, GAPDHrev  TCGCTCCTGGAAGATGGTGAT. Relative expression was normalized from GAPDH. Results were expressed in arbitrary units (A.U).
